# Seasonal and Nutrient Supplement Responses in Rumen Microbiota Structure and Metabolites of Tropical Rangeland Cattle

**DOI:** 10.3390/microorganisms8101550

**Published:** 2020-10-08

**Authors:** Gonzalo Martinez-Fernandez, Jinzhen Jiao, Jagadish Padmanabha, Stuart E. Denman, Christopher S. McSweeney

**Affiliations:** 1 Agriculture and Food, CSIRO, St Lucia, QLD 4067, Australia; gonzalo.martinezfernandez@csiro.au (G.M.-F.); Jagadish.Padmanabha@csiro.au (J.P.); stuart.denman@csiro.au (S.E.D.); 2 CAS Key Laboratory of Agro-ecological Processes in Subtropical Region, Institute of Subtropical Agriculture, The Chinese Academy of Sciences, Changsha 410125, China; jjz@isa.ac.cn

**Keywords:** rumen, microbiota, nitrogen supplementation, tropical rangelands, seasonal effect

## Abstract

This study aimed to characterize the rumen microbiota structure of cattle grazing in tropical rangelands throughout seasons and their responses in rumen ecology and productivity to a N-based supplement during the dry season. Twenty pregnant heifers grazing during the dry season of northern Australia were allocated to either N-supplemented or un-supplemented diets and monitored through the seasons. Rumen fluid, blood, and feces were analyzed before supplementation (mid-dry season), after two months supplementation (late-dry season), and post supplementation (wet season). Supplementation increased average daily weight gain (ADWG), rumen NH_3_–N, branched fatty acids, butyrate and acetic:propionic ratio, and decreased plasma δ^15^N. The supplement promoted bacterial populations involved in hemicellulose and pectin degradation and ammonia assimilation: *Bacteroidales BS11*, Cyanobacteria, and *Prevotella* spp. During the dry season, fibrolytic populations were promoted: the bacteria *Fibrobacter*, Cyanobacteria and Kiritimatiellaeota groups; the fungi *Cyllamyces*; and the protozoa *Ostracodinium*. The wet season increased the abundances of rumen protozoa and fungi populations, with increases of bacterial families *Lachnospiraceae*, *Ruminococcaceae*, and *Muribaculaceae*; the protozoa *Entodinium* and *Eudiplodinium*; the fungi *Pecoramyces*; and the archaea *Methanosphera*. In conclusion, the rumen microbiota of cattle grazing in a tropical grassland is distinctive from published studies that mainly describe ruminants consuming better quality diets.

## 1. Introduction

The northern Australia beef industry is based on extensive grazing in dry tropical rangelands such as the grazing systems in Africa and South America. This environment is dominated by a short wet and long dry season, where undernutrition is the major constraint for the majority of the year and coincides with the long dry winter [1]. Animals typically lose body condition during the dry season and energy reserves are further depleted in late pregnancy or during lactation due to the high nutritional demands of these physiological states [2].

Typically, several management strategies are used in this environment to alleviate the seasonal consequences on the animals [2]. Pregnant animals are provided with feed supplements during the dry season and calving is timed for the late dry and early wet season, when higher quality pasture becomes available. Beef enterprises commonly use supplements that contain different levels of macronutrients such as crude protein, sulfur, and phosphorus to correct the nutritional deficiencies. Due to cost factors, these nutrients are usually in an inorganic form (e.g., crude protein as urea/ammoniated salts; sulfur as elemental sulfur or sulfate) that can be used by the rumen microorganisms.

Positive growth responses to N supplements for cattle consuming low quality tropical forage have been reported for pen-trials [3,4,5,6]. Studies in grazing cattle during the dry season have shown increases in re-conception rates in first lactation heifers supplemented with inorganic macronutrients [7], a lower weight loss in steers supplemented with urea [8,9], and growth increases in young animals supplemented with urea and molasses [10,11]. While the nutritional benefits of these supplements are well documented, there is little information about rumen metabolism, the composition of the rumen microbial community, and their responses to these nutrients in tropically adapted grazing cattle, which represent a large proportion of the global population of ruminants. In addition, there is no information on the rumen microbial changes due to the improvement of pasture quality between seasons, and the majority of the published studies [12] have characterized the rumen microbial communities from cattle fed temperate diets under non extensive conditions.

In this study, we investigated the changes that occur in rumen metabolism and microbiota structure and productivity of pregnant heifers provided with a commercial lick-block supplement in the dry season, and characterized the diversity of the rumen microbial populations (bacteria, protozoa, fungi, and archaea) and rumen fermentation changes of cattle grazing in tropical rangeland during the dry and wet seasons.

## 2. Materials and Methods

### 2.1. Experimental Design and Sampling

The experimental protocol complied with the Australian Code for the Care and Use of Animals for Scientific Purposes (eighth edition, 2013) and was approved by the Commonwealth Scientific and Industrial Research Organization (CSIRO) Wildlife and Large Animal Ethics Committee, application number 2016-12.

Twenty pregnant crossbred heifers (*Bos indicus x Bos taurus*, average body weight 399.8 kg) were maintained in a 1200 ha paddock at a cattle station located on the Barkley Tableland in the Northern Territory of Australia, with the grassland composition consisting mainly of Hoop Mitchell (*Astrebla elyminoides*) and native millet (*Panicum decopositum*) in the dry season, silky browntop (*Eulalia aurea*) and feathertop wiregrass (*Aristida latifolia*) in the late dry season, and Flinders grass (*Iseilema spp*) during the wet season. Seasonal nutritional value of the Barkly Tableland pasture grasses are as follows: Dry season: crude protein between 2–4 %, neutral detergent fiber (NDF) between 68–72%, acid detergent fiber (ADF) between 39–42%, and hemicellulose between 23–30%; and Wet season: crude protein between 6–10%, NDF between 64–70%, ADF between 37–45%, and hemicellulose between 27–29% [13,14] (CSIRO un-published data).

Animals were selected for the same pregnancy stage (5.5 months pregnant ± 2 weeks) and randomly allocated to two groups: Rumevite^®^ block (supplemented, *n* = 10; body weight mean: 401.3 kg) (Table S1) or control (un-supplemented, *n* = 10; body weight mean: 398.2 kg). Groups were not significantly different prior to supplementation (Table S2). During the dry season, animals were auto drafted daily to their respective treatment (supplemented or un-supplemented) at the paddock water points using an automated draft system with a radio frequency identification (RFID) reader that identified the animal RFID tag. The treatment was placed on a platform that recorded the weight of the supplement product and the weight change that occurred while each individual animal was consuming the supplement. Animals were identified by a RFID reader that recorded the animal RFID tag and the time spent with the supplement. The supplementation started in mid-August 2016 and stopped in December 2016 (beginning of the wet season), when calving started. Calves were kept with their mothers (lactating) during the trial and were not monitored or sampled.

Samples were collected at pre-supplementation (August 2016, mid-dry season; mid-pregnancy), after two months supplementation (October 2016, late-dry season; late-pregnancy), and post supplementation (February 2017, wet season; lactation) (Figure S1). Each collection started at the same time after moving the animals from the paddock to the yards. Body weight was recorded at each sampling point to calculate ADWG, and rumen fluid, blood, and fecal samples were collected to study the rumen microbial and fermentation profile, plasma metabolites, and fecal nitrogen, respectively. Rumen fluid samples were collected by esophageal intubation, samples were immediately frozen using dry ice, and stored at −20 °C for ruminal fermentation metabolites or at −80 °C prior to DNA extractions for microbial community composition.

Blood samples from all animals were collected by jugular venipuncture using a 10 mL blood Vacutainer tube (BD, Sydney, Australia) containing sodium heparin for plasma and a 10 mL blood Vacutainer tube coated with silica for serum. Blood samples for plasma were immediately placed on ice and blood samples for serum were kept for 1 h at room temperature before placing on ice prior to centrifugation. Both blood samples were centrifuged (2500 rpm for 20 min at 4 °C) to separate the plasma and the serum, which were stored at −80 °C for blood urea nitrogen (BUN) analysis from the serum and N isotopic natural abundance analysis from plasma.

### 2.2. Laboratory Analysis

Fecal samples were freeze-dried in an Epsilon 2–6D LSC plus freeze dryer (Martin Christ, Osterode, Germany) and ground to 1 mm size in an Ultra Centrifugal Mill ZM200 (Retsch, Haan, Germany). Nitrogen fecal content was determined by near-infrared spectroscopy (NIRS) (NIRSystems FOSS 6500) at the CSIRO Floreat laboratory (Floreat, WA, Australia). ISI (Infrasoft International) software NIRS 3 (Version 3.10, Port Matilda, PA, USA) was used for all spectral analyses, data manipulation, and spectra calibrations. The calibration equations used for fecal nitrogen by near-infrared spectroscopy (FNIRS) predictions were developed for cattle grazing tropical/subtropical pastures [15,16].

Concentrations of volatile fatty acids (VFAs) (acetate, propionate, n-butyrate, iso-butyrate, iso-valerate, and n-valerate) were measured by gas chromatography as described by Gagen et al. [17]. Iso-valerate (3-methyl butyrate) includes 2-methylbutyrate, which co-elutes.

The ammonia–N rumen concentration and BUN were determined by using the previously published colorimetric method [18].

### 2.3. Natural Abundance Analysis of Nitrogen Stable Isotopes

Plasma samples were thawed at 4 °C overnight and their protein fraction was isolated by precipitation with sulfosalicylic acid (200 µL of 1 g/mL into 2 mL of sample). After 1 h of incubation at 4 °C and centrifugation (4500× *g* for 20 min at 4 °C), the supernatant and pellet were separated [19]. The pellet was rinsed three times with MilliQ water and then freeze-dried. The N stable isotopic composition (δ^15^N, i.e., natural relative abundance of the rare stable isotope of N) of plasma protein was determined using a Carlo Erba NA1500 elemental analyzer coupled to a Delta V plus isotope-ratio mass spectrometer via a Conflo IV (Thermo Scientific, Waltham, MA, USA). Stable isotope results were corrected via a three-point calibration using international primary reference standards. A secondary reference was included after every 12 samples to monitor any instrument drift. Nitrogen was quantified by comparison of peak areas against a response calibration curve.

Results are expressed using the delta notation according to the following equation:

δ^15^N = ((*R*_sample_/*R*_standard_) – 1) × 1000 (1)

where *R*_sample_ and *R*_standard_ are the N isotope ratio between the heavier isotope and the lighter isotope (^15^N:^14^N) for the sample being analyzed and the internationally defined standard (atmospheric N_2_, *R*_standard_ = 0.0036765), respectively, and δ is the delta notation in parts per 1000 (‰) relative to the standard.

### 2.4. DNA Extractions and Illumina MiSeq Sequencing

DNA extractions from rumen samples were performed as described by Martinez-Fernandez et al. [20]. The 16S rRNA, ITS, and 18S rRNA genes were used to characterize the microbial populations in the rumen for both bacteria (v4 region) [21] and archaea (v6-v8 region) [22,23], fungi [24], and protozoa [25], respectively. Each DNA sample was amplified using the specific primers (Table S3) and a unique barcode combination as described by de Carcer et al. [26]. Amplification products were visualized by performing gel electrophoresis. Product quantities were calculated, and an equal molar amount of each target product was pooled. The pooled target products were run in a 1.5% agarose gel and bands were visualized and excised under blue light trans-illumination. The amplicons were gel purified with a QIAquick Gel Extraction Kit (Qiagen, Hilden, Germany) prior to submission for 2 × 250 bp Illumina MiSeq sequencing (Macrogen Inc., Seoul, Korea).

Paired-end short-read sequence data generated on the Illumina MiSeq was processed using the USEARCH package [27]. De-multiplexed paired-end sequences were first merged prior to sequence quality filtering, followed by denoising (error correction), chimera checking, and clustering of sequences to Amplicon sequence variants (ASVs) [28]. Analysis of microbiota diversity and identification of ASVs significantly altered by supplementation or seasons was performed in R following the compositional data analysis using mixOmics [29] and the workflow developed by Gloor et al. [30] using packages Phyloseq [31], CoDaSeq, propr, vegan, and ALDEx2. Discriminative ASVs were selected at false discovery rate (FDR) ≤ 0.05 (Benjamini–Hochberg false discovery rate) and at the absolute expected standardized effect size >1. Taxonomic classification of bacterial ASVs was done using the IDTAXA algorithm implemented in the DECIPHER R package against the SILVA SSU r132 training set [32]. Taxonomic classifications of methanogenic archaea, ciliate protozoa, and anaerobic fungi were assigned using the assign Taxonomy implemented in the DADA2 R package for the RIM-DB database [33], intestinal ciliate protozoa [34], and the Anaerobic Fungal ITS1 database V3.4 [35], respectively. Two animals were excluded from the microbiota analysis due to sequencing issues.

### 2.5. Quantitative PCR Analysis

The DNA samples were used as templates for quantifying the abundance of anaerobic rumen fungi and protozoa populations. The primers and assay conditions used for fungi and protozoa, respectively, were previously published by Denman and McSweeney [36] and Sylvester et al. [37]. Quantitative PCR (qPCR) analyses were run in quadruplicate from one DNA extraction on an Applied Biosystems™ ViiA™ 7 Real-Time PCR System (Thermo Fisher Scientific Inc., Waltham, MA, USA). Assays were set up using the SensiFAST SYBR^®^ Lo-ROX reagents (Bioline Pty Ltd., Eveleigh, Australia). A total rumen microbial DNA template concentration of 50 ng was used for each assay under the following cycle conditions: one cycle of 50 °C for 10 s and 95 °C for 2 min 30 s for initial denaturation, forty cycles at 95 °C for 15 s, and 60 °C for 1 min for primer annealing and product elongation. Fluorescence detection was performed at the end of each annealing and extension step. Amplicon specificity was performed via dissociation curve analysis of PCR end products by raising the temperature at a rate of 0.05 °C/s from 60 to 95 °C. Changes in targeted populations were calculated using a relative quantification calculation and the 2-∆∆Ct method, with the dry season period or control group (un-supplemented animals) used as the calibrator and total bacterial Ct (cycle threshold) values used as the reference value [36,38].

### 2.6. Statistical Analyses

Rumen fermentation parameters, fecal nitrogen, blood metabolites, qPCR data, and animal performance parameters were analyzed as a univariate model using the GLM procedure of SPSS (IBM Corp., version 21.0, Armonk, NY, USA), with the animal as the experimental unit. The effect of treatment was analyzed for all the parameters in the late-dry season. Effects were considered significant at *p* ≤ 0.05 and *p*-values between 0.05 and 0.10 were considered as a trend. When significant differences were detected, differences among means were tested with the least significant difference (LSD) comparison test. Data from the mid-dry and wet seasons were analyzed separately as a repeated-measures analysis using the GLM procedure of SPSS (IBM Corp., version 21.0, Armonk, NY, USA), with the animal as the experimental unit to study the seasonal effect.

## 3. Results

### 3.1. Rumen Fermentation, Blood, and Fecal Parameters

The supplement effects in the late-dry season are shown in Table 1. Supplemented animals showed significant (*p* ≤ 0.05) increases in nitrogen intake (23 g/d), ADWG (0.67 vs. −0.37 kg/d), and animal body weight (437 vs. 379 kg) compared with the control group. BUN and fecal nitrogen did not change between the supplemented and un-supplemented animals. However, a significantly (*p* ≤ 0.05) lower δ ^15^N in plasma was detected in supplemented animals compared with the un-supplemented group (5.44 vs. 7.11 ‰, respectively).

Regarding rumen fermentation parameters, significant increases (*p* ≤ 0.05) in proportions of acetate and butyrate and a decrease in propionate were found with the supplemented animals compared with the control group. A numerical but non-significant increase in rumen ammonia was observed in the supplemented animals.

The seasonal effects on rumen fermentation parameters, blood urea nitrogen, fecal nitrogen, and body weight are shown in Table 2. A significantly (*p* ≤ 0.05) lower concentration of rumen ammonia (11.9 mg/L) and BUN (2.79 mg/ 100 mL) was detected in the mid-dry season compared with the wet season (70.2 and 9.04 mg/100 mL, respectively). Interestingly, total VFA concentration was significantly higher (84.0 mM vs. 64.2 mM) in the dry compared with the wet season. Regarding the individual VFA profile, a higher (*p* ≤ 0.05) concentration of branched fatty acids and valerate, and lower (*p* ≤ 0.05) propionate were found in animals grazing during the wet season. A significant increase in fecal N reflecting pasture quality was reported in the wet compared with the dry season (0.97 vs. 2.12 %).

### 3.2. Rumen Microbial Community

#### 3.2.1. Supplementation Response

Alpha diversity analysis of the rumen bacteria and protozoa populations revealed a significant increase in the number of Chao1 estimated ASVs as well as increased Shannon and Simpson diversity indexes for the rumen bacteria only in the supplemented group when compared to the un-supplemented animals in the late-dry season (Figure S2a,c). However, alpha diversity analysis of rumen methanogenic archaea and fungi showed no differences between un-supplemented and supplemented animals (Figure S2b,d).

The supervised multivariate approach using sparse partial least squares discriminant analysis (sPLS-DA) confirmed that the major drivers of the variance in the late-dry season relates to the nitrogen based supplement, showing a clear separation between supplemented and un-supplemented groups for rumen bacteria (Figure S3a), and less clear or no separation for archaea, protozoa, and fungi, respectively (Figure S3b–d).

**Figure 1:**
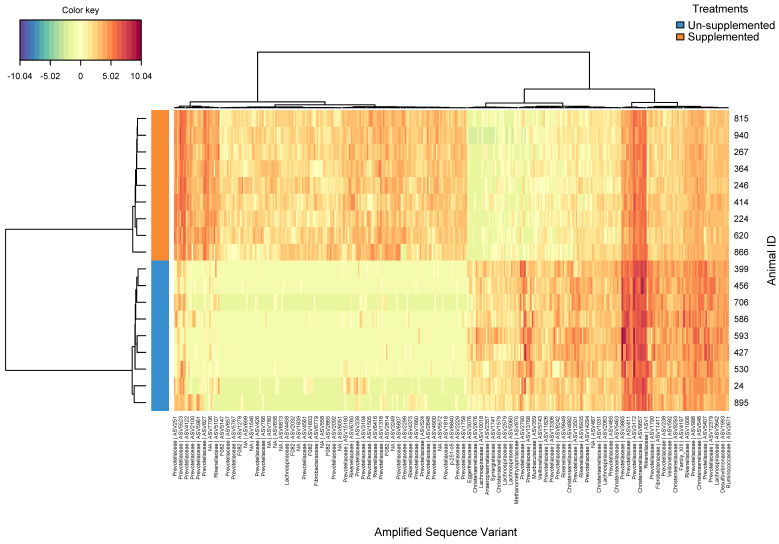
Clustering analysis using a heatmap based on the bacterial ASVs significantly different from un-supplemented and supplemented animals during the late-dry season.

The clustered image map for the rumen bacterial ASVs that were significantly different (FDR ≤ 0.05) in the late-dry season showed a distinctive microbial signature for each experimental group (Figure 1), with 377 ASVs identified to be significantly different (FDR < 0.05) between both groups (Supplementary File S1). Most notably, in the supplemented group were detected increases in ASVs associated with novel groups of Bacteroidetes from the families *F082* and *BS11*, along with Cyanobacteria (*Melainabacteria*) and certain *Prevotellaceae*. Conversely, the center log ratio of ASVs assigned to Synergistetes, *Veillonellaceae*, *Succinivibrionaceae, Lachnospiraceae*, *Rikenellaceae*, and some *Prevotellaceae* were higher in the un-supplemented group. Regarding the rumen methanogenic archaea, ASVs assigned to *Methanobrevibacter gottschalkii* were significantly (FDR ≤ 0.05) associated with supplemented animals (Supplementary File S2).

On the other hand, no significant differences were detected in anaerobic fungi and protozoa ASVs or abundances assessed by qPCR for fungi (Figure S4) in supplemented animals compared with the un-supplemented group in the late-dry season. However, a trend (*p* = 0.098) was found in protozoa populations abundances, with a 0.5 fold increase in supplemented treated animals.

#### 3.2.2. Seasonal Effect

Alpha diversity analysis of the rumen microbial populations revealed a significant increase in Chao1 estimated ASVs as well as Shannon and Simpson diversity indexes for protozoa in the wet season compared to the mid-dry season (Figure S5c), and a significant decrease in Chao1 estimated ASVs as well as Shannon and Simpson diversity indexes for bacteria in the wet season. However, no significant differences were detected in Chao1 estimated ASVs for rumen methanogenic archaea, and anaerobic fungi communities (Figure S5b,d).

**Figure 2:**
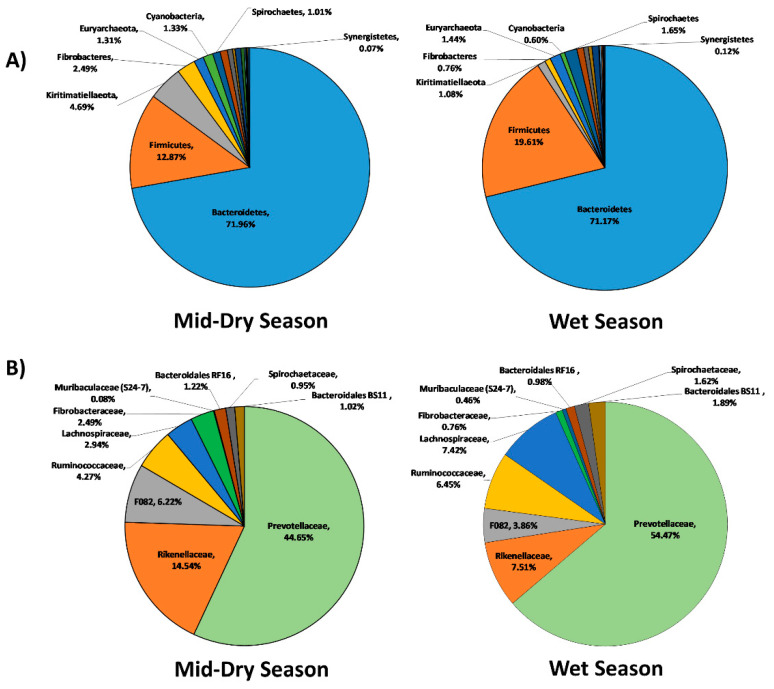
Taxonomic composition of the rumen bacteria community at the phylum level (**A**) and family level (**B**) for the mid-dry and wet seasons in un-supplemented animals.

Analysis of the rumen bacterial microbiota showed a shift in the relative abundance at the phylum level when animals were grazing in the mid-dry and wet season (Figure 2A). An increase in the sequences assigned to the Firmicutes, Synergistetes, and Spirochaetes phyla and decreases in Fibrobacteres, Kiritimatiellaeota, and Cyanobacteria were observed when animals were grazing in the wet season. At the family level (Figure 2B), increases in sequences classified to *Prevotellaceae, Ruminococcaceae, Lachnospiraceae, Muribaculaceae (S24-7), Spirochaetaceae,* and *Bacteriodales BS11* group, and decreases in *Rikenellaceae*, the *F082* group, and *Fibrobacteraceae* were associated with animals grazing in the wet season.

**Figure 3:**
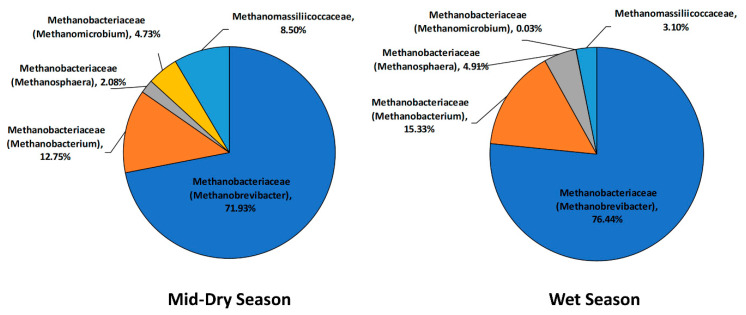
Taxonomic composition of the rumen archaea community at family and genus level for the mid-dry and wet seasons in un-supplemented animals.

The archaea community shifted at the genus level (Figure 3) with increases in reads assigned to *Methanobacterium*, *Methanosphaera*, and *Methanobrevibacter*, and decreases in *Methanomicrobium spp.* and *Methanomassiliicoccaceae* family reads in the wet season.

**Figure 4:**
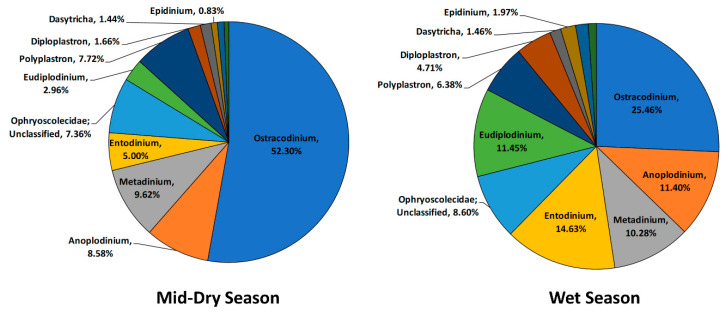
Taxonomic composition of the rumen protozoa community at genus level for the mid-dry and wet seasons in un-supplemented animals.

The rumen protozoa taxonomy structure also changed between seasons (Figure 4), with increases in reads assigned to *Eudiplodinium*, *Entodinium*, *Metadinium*, *Anoplodinium*, and *Diploplastron* species and decreases in *Ostracodinium* and *Polyplastron* associated with the wet season animals.

**Figure 5:**
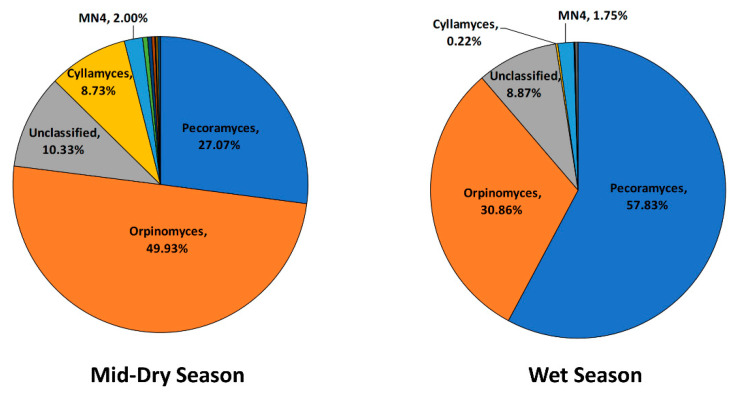
Taxonomic composition of the rumen fungi community at the genus level for the mid-dry and wet seasons in un-supplemented animals.

Regarding the fungal taxonomy structure (Figure 5), during the wet season, there was an increase in reads assigned to the *Pecoramyces* genus and decreases in the *Cyllamyces* and *Orpinomyces* species.

The sPLS-DA produced a clear separation between animals grazing in the mid-dry and wet season for rumen bacteria, methanogenic archaea, protozoa, and anaerobic fungi populations, mainly driven by the changes in diet (Figure S3a–d).

**Figure 6:**
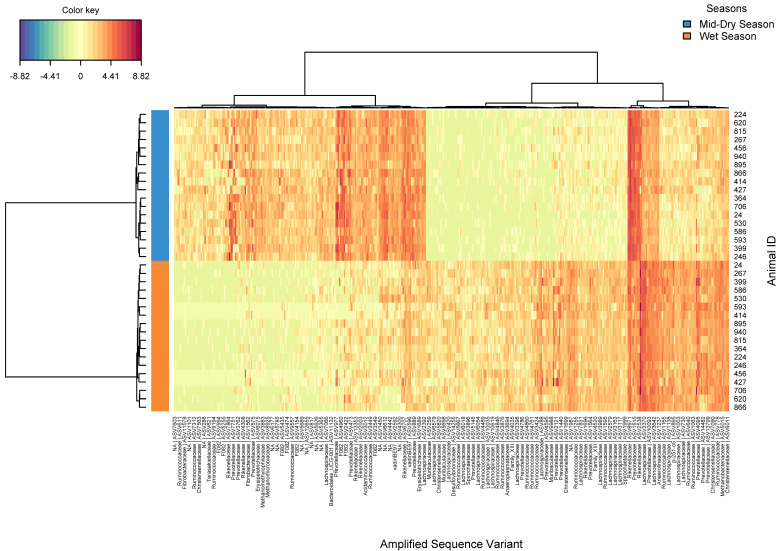
Clustering analysis using heatmap based on the bacterial ASVs significantly different from animals grazing in the mid-dry and wet seasons.

The clustered image map of the rumen bacteria ASVs that differed significantly (FDR ≤ 0.05) between seasons showed distinctive signatures for each season (Figure 6). A total of 454 ASVs were identified to be significantly different between both seasons (Supplementary File S3). Distinctively, in the wet season, ASVs assigned to the families *Lachnospiraceae* (particularly *Butyrivibrio* genus), *Ruminococcaceae, Muribaculaceae (S24-7)*, the *Bacteroidales BS11* group, and *Veillonellaceae* were increased, while ASVs associated with *Fibrobacter*, *Rikenellaceae RC9*, *Succiniclasticum,* the Bacteroidetes *F082* group, and the phyla Kiritimatiellaeota and Cyanobacteria (*Melainabacteria*) were decreased, reflecting the shift to higher quality pasture and animal physiological changes (pregnant vs. lactating).

Regarding the methanogenic archaea community, two ASVs classified to *Methanosphaera* spp. were significantly increased in the wet season, while two ASVs within the *Methanomassiliicoccaceae* family and *Methanomicrobium mobile* were associated with the mid-dry season animals (Supplementary File S4).

The changes in the rumen protozoa structure between seasons were mainly associated with a significant (FDR ≤ 0.05) increase of two AVSs assigned to the genera *Entodinium* and *Eudiplodinium* and a decrease of two AVS associated with *Ostracodinium* in the wet season grazing animals (Supplementary File S5).

The characterization of the rumen anaerobic fungi showed significant (FDR ≤ 0.05) increases in 10 ASVs assigned to *Orpinomyces* and three ASVS to *Pecoramyces*, while eight ASVs belonging to *Cyllamyces* and three to *Orpinomyces* significantly decreased in the wet season animals compared to when they were grazing in the mid-dry season (Supplementary File S6).

Finally, the qPCR analysis of rumen anaerobic fungi and protozoa communities showed a significant (*p* ≤ 0.05) increase (6 and 8-fold, respectively) of these populations in animals grazing during the wet season compared to the mid-dry season (Figure S6).

## 4. Discussion

### 4.1. Productivity and Rumen Fermentation Responses

Tropical rangeland grasses rarely provide the minimum level of nutrients, particularly during the dry season, limiting pasture intake and feed digestibility for the grazing cattle [39]. Tropical grasses are typically low in N and metabolizable energy, with a faster maturation and higher fiber component than temperate grasses [9]. During the dry season, the main limiting nutrient in the pasture is N, which is critical (among other nutrients) for adequate microbial growth and fiber digestibility. Around 60–90% of dietary protein is converted to ammonia in the rumen with 50–70% of rumen bacterial protein originating from this source [40,41]. A rumen concentration of 50 mg NH_3_–N/L has been identified as the optimal level to maximize microbial protein production [42,43], and the limiting concentration has been suggested to be around 20 mg NH_3_–N/L based on in vitro analysis [42]. However, studies with cattle on tropical roughage-based diets have recommended that supplementation with non-protein nitrogen should be performed when rumen ammonia concentration falls below 45 mg/L [44].

As expected, supplementation with non-protein nitrogen to cattle grazing low quality tropical pastures had a remarkable response on body weight and shifted the rumen fermentation profile. The ADWG of the supplemented pregnant heifers increased over 0.500 kg per day, which is greater than that in previous published studies [2,9,10,11,39,45]. However, these studies reported a less acute loss of weight, maintenance, or increases up to 0.300 kg per day in cattle supplemented with urea and other micronutrients (e.g., sulfur) during the dry season. A deficient level of rumen ammonia–N and BUN was observed in all animals in the mid-dry season, with an improvement during supplementation. However, this increase was still below the recommended level of 45–50 mg NH_3_–N/L [42,44] for the maximum rate of fermentation and microbial protein production. Regarding BUN, a concentration between 8 to 10 mg/dL is considered to be an optimum balance between energy intake and digestible protein [46,47]. During the wet season, both parameters increased to sufficient levels (over 50 mg/L and 9 mg/dL for rumen ammonia and BUN, respectively) due to the improvement in the quality and dietary crude protein of the pasture. Interestingly, total VFA concentration was higher in the dry compared with the wet season, which might be influenced by a decrease in the rumen volume due to the fetal growth (as heifers were pregnant at the dry season) or differences in absorption and passage rate between the seasons.

FNIRS has been reported to be a cost effective and rapid tool to predict the quality of the pasture consumed by cattle [8,9]. However, the technique cannot discriminate between animals receiving a urea based supplement and those not supplemented [9] under the same grazing regime. Our results are in line with these findings, showing an increase in fecal N in the wet season, when major changes to the quality of the pasture occurred, and no differences in FNIRS between supplemented and un-supplemented animals while grazing together in the dry season. Aside from this limitation, FNIRS is still a relevant tool that beef enterprises can use for making feed management decisions between seasons.

The natural abundance of N isotopes in animal tissues relative to the diet has been proposed as a proxy for N use efficiency in ruminants [48] and it could be used as a biomarker to evaluate N use by the animal when is not possible to measure the feed intake or diet composition [49]. The current study showed a lower plasma δ ^15^N in the supplemented animals, which is an indication of the use by the animal of different N components in the diet, as the supplement is naturally depleted in δ ^15^N (−1.16‰) compared to the main dietary components (forage: 1.63‰). Thus, the plasma δ ^15^N might be used as a biomarker to rapidly identify those animals consuming N supplements, leading to a more efficient management of the herds. In addition, a more efficient use of N might have occurred in the supplemented animals as they were in an anabolic state, while the un-supplemented animals were losing weight. However, further research needs to be done to confirm this.

### 4.2. Rumen Microbial Responses

The rumen microbiota is comprised of bacterial, archaeal, protozoal, fungal, and phage populations, which play important roles in rumen function and are modulated primarily by diet composition. This study has characterized for the first time the rumen microbial community composition in grazing cattle under supplementation in dry tropical rangelands of northern Australia.

#### 4.2.1. Bacteria Community

Most of the ruminant studies [12] where temperate diets are common have reported greater relative abundance of Firmicutes and lower Bacteroidetes than the current study. However, our observation involving cattle grazing in dry tropical rangelands agrees with the high ratio reported by McCann et al. [50] in Brahman cattle fed Coastal Bermuda-grass and other studies using tropical adapted cattle fed a tropical hay [20,51]. Therefore, a consistent observation is emerging that the bacterial microbiota of ruminants consuming a diet high in lignocellulose is dominated by Bacteroidetes, unlike diets comprised of more readily fermentable carbohydrates.

Many of the bacterial ASVs detected in this study align with un-cultured species, but recent studies using metagenomic sequencing and assembly techniques have produced metagenomic assembled genome reconstructions for representatives of the *Muribaculaceae* (S24-7), *Bacteroidales BS11*, and Cyanobacteria *(Melainabacteria)* family, allowing for functional predictions to be assigned [52,53,54]. All are considered hemicellulose degraders and produce acetate and butyrate as their major end products, respectively. The variation in the relative abundance of these species between the seasons is likely being driven by the change in the quality of the diet and their ability to degrade the hemicellulose content of the plant. The *Muribaculaceae* (S24-7) family is associated with the gastrointestinal system of homeothermic animals and possesses enzymes able to degrade hemicellulose and pectin [53], which is usually higher during the wet season. Another interesting observation was the greater relative abundances of Fibrobacteres, Kiritimatiellaeota, and Cyanobacteria during the dry season when the fiber content of the diet is highest. These bacterial populations appear to be positively correlated with the NDF and ADF content of the diet [55]. Fibrobacteres phylum is composed of a single cellulolytic genus; Kiritimatiellaeota has been suggested to be involved in fiber degradation; and the rumen associated Cyanobacteria group are polysaccharolytic organisms [56].

The *Bacteroidales BS11* group and Cyanobacteria (*Melainabacteria*) would appear to perform better in the presence of nitrogen supplementation and correlate with higher ratios of acetate and butyrate production in these animals. *BS11* species have been observed to increase in numbers in moose when the diet is composed of highly indigestible material (bark) [52], while in humans, the Cyanobacteria (*Melainabacteria*) group is increased with a higher plant based diet [54]. On the other hand, some *Prevotella* groups have also responded positively to nitrogen supplementation; this bacterial group is also hemicellulolytic, while using peptides and ammonia as nitrogen sources [57] and can adapt to low nitrogen environments by regulating the expression of genes involved in nitrogen assimilation [58]. Regarding the higher proportional propionate concentration observed in the un-supplemented animals in the dry season, this coincided with increases in some members from the families *Veillonellaceae* and *Prevotellaceae* that are also capable of producing propionate as a major fermentation end-product [59,60]. The increase in propionate might be explained by a decrease in its absorption rate through the rumen wall or a decrease in the production of the other VFAs.

#### 4.2.2. Archaea Community

Rumen methanogenic archaea play a specific role in the rumen ecosystem, producing methane mainly through hydrogenotrophic and methylotrophic pathways. The global rumen census [12] reported that around 75% of archaea found in the rumen were hydrogenotrophic (*Methanobrevibacter* being the most dominant group) and 20% methylotrophic (mainly *Methanosphera* and *Methanomassiliicoccaceae* groups). Although our study found more than 70% of archaea sequences were assigned to *Methanobrevibacter*, the methylotrophic methanogens were found to comprise less than 10% in animals grazing tropical roughages. Interestingly, the *Methanobacterium* genus, which has been reported to be a minor methanogen population in the rumen [12,61], was the second most dominant group (12–15%) in our study. *Methanobacterium* spp, which is able to grow and produce methane from formate and H_2_/CO_2_, [62], were also observed as dominant methanogens in the rumen of roughage consuming buffalo [63], compared to other methanogens.

The only significant change in methanogen diversity during the supplementation period was the increase of ASVs assigned to *Methanobrevibacter gottachlkii,* a hydrogenotrophic methanogen. This may have been a direct response to the urea supplementation as *Methanobrevibacter spp.* compete with bacteria for ammonia as a nitrogen source [64,65] for protein synthesis. However, species specific inter-relationships between methanogens and other organisms in the rumen could also be implicated.

Regarding the seasonal effects, the major changes in methanogen were shifts in ASVs assigned to the methylotrophic methanogens *Methanomassiliicoccaceae* (methylamine and methanol utilizer) and *Methanosphera* (methanol utilizer) and the hydrogenotrophic *Methanomicrobium mobile* [66]. The *Methanomassiliicoccaceae* family was more dominant in the dry season than *Methanosphera,* which might reflect a greater concentration of methylamines in the pasture. Methylamines are generated from plant phosphatidylcholine degradation, while methanol is produced from the demethoxylation of dietary pectins [66]. During the wet season, *Methanosphaera* was the dominant methylotroph, which might indicate an increase in pectin on the diet. Pectin has been reported to be inversely related to the level of lignification of the plant wall [67], which agrees with the forage characteristic during the wet season. The changes in methylotrophic methanogens might also be linked to the increase in protozoa abundances observed in the wet season, as methanol is the major end-product of the enzymatic degradation of pectin by rumen protozoa [68]. In addition, some rumen bacteria from the genera *Butyrivibrio*, *Prevotella,* and *Lachnospira* [66] are also able to degrade pectin and release methanol. ASVs assigned to these bacteria genera increased in the wet season, which supports this hypothesis.

#### 4.2.3. Protozoa Community

Ciliate protozoa populations differ with changes in diet and can represent up to 50% of the total rumen biomass [68]. They perform important functions such as fiber degradation, oxygen scavenging, regulation of the bacterial protein turnover through bacteria predation as well as influencing methane emissions [69,70]. According to the global rumen census report [12], *Entodinium* and *Epidinium* dominate the rumen ecosystem, representing 54.7% of protozoal sequence data. In the current study, *Ostracodinium* was found to be the most dominant protozoa genera in all of the samples (20–45%), implying the important role of these protozoa in fiber degradation in grazing systems, as members of this group possess a high cellulolytic activity [70].

A particularly interesting observation during the wet season was the marked increase in protozoa numbers and the shift from *Ostracodinium* to *Entodinium* and *Eudiplodinium*. These changes are likely to be attributed to a higher-quality diet and more rapidly degradable carbohydrates available with concurrent changes to the bacterial biomass, which might influence bacterial predation by protozoa such as *Entodinium* [71]. Although little is known about the seasonal effects on the rumen protozoa community, a similar trend was reported in previous published studies in ruminants [68,72,73,74]. These showed an increase in protozoa counts during summer, with the greatest increase accounted for by the *Entodinium* genus, and a decrease in total counts in winter when the pasture quality declined. A higher percentage of protozoa groups that can utilize the lignified forage were also found such as occurred with *Ostracodinium* in the current study.

Rumen defaunation (elimination of protozoa from the rumen) has been proposed as a strategy to reduce methane emissions, increase the duodenal flow of microbial protein, and improve the efficiency of microbial protein synthesis in ruminants [69,70]. However, its adoption might be detrimental for animals, particularly in grazing systems rich in poor-quality forage such as the dry tropical rangelands. This would likely be the result of decreases in fiber digestibility and feed intake from the protozoa-free ruminants, as reported by a recently published meta-analysis [69].

#### 4.2.4. Fungi Community

Anaerobic fungi play important roles in lignocellulose decomposition in the rumen through physical penetration and secretion of cell–wall-degrading enzymes. The metabolites produced during this process are mainly hydrogen, formate, and acetate [75]. Currently, only eighteen anaerobic fungi genera have been isolated and identified from herbivores [76,77,78]. In our study, on tropical rangelands pastures, the most dominant genera through the seasons were *Cyllamyces*, *Orpinomyces*, and *Pecoramyces*. A shift in certain *Orpinomyces* spp and an increase in *Pecoramyces* genus were the main changes during the wet season, which are likely to be linked to the marked improvement in forage quality and rapid pasture growth. The higher proportion of certain *Orpinomyces* and *Cyllamyces* groups during the dry season relative to the wet might be due to the lower pasture quality, as *Cyllamyces* plays an important role in the degradation of poor quality feed [79]. In addition, Couger et al. [80] identified the *Orpinomyces* genus as a very efficient degrader of multiple types of lignocellulosic biomass. However, these findings need to be considered carefully due to several limitations in relation to the characterization of the anaerobic fungi community: First, the sampling method used in the current study (stomach intubation) likely under-represents and underestimates the anaerobic fungi population, as it mainly collects the liquid phase of the rumen contents; and second, the current anaerobic fungi database is still incomplete and small, which might result in the failure to taxonomically identify some organisms correctly [81].

## 5. Conclusions

This study showed that the rumen microbiota of cattle grazing a dry tropical grassland is different from most of the published studies that have focused primarily on ruminants consuming better quality diets. The distinctive features included the bacterial community being dominated by members of the Bacteroidetes; methanogens affiliating with the *Methanobacterium* genus were more highly represented than usual; the protozoal population was characterized by a large proportion of the cellulolytic *Ostracodinium* spp; and the anaerobic fungal population was comprised mainly of the fibrolytic genera *Cyllamyces*, *Orpinomyces*, and *Pecoramyces*. Supplementation of inorganic nutrients (e.g., urea) in a low nitrogen environment, characteristic of the dry tropical rangelands, enhanced rumen fermentation and promoted bacterial populations involved in hemicellulose and pectin degradation and ammonia assimilation: *Bacteroidales BS11*, Cyanobacteria (*Melainabacteria*), and *Prevotellaceae*. This cost-effective supplementation strategy also improved the animal weight gain during environmental conditions that normally lead to catabolic states. During the dry season, the increase in lignocellulose and decrease in N content changed the rumen microbiota structure, promoting populations that have been identified as fiber degraders such as bacteria from *Fibrobacter*, Cyanobacteria, and Kiritimatiellaeota groups; the fungi *Cyllamyces;* and the protozoa *Ostracodinium*. The improvement in quality and biomass of the diet during the wet season increased the abundances of rumen protozoa and fungi populations and significantly shifted the rumen microbiota structure, with increases in the bacterial families *Lachnospiraceae*, *Ruminococcaceae,* and *Muribaculaceae*; the protozoa *Entodinium* and *Eudiplodinium;* and the fungi *Pecoramyces*. The major change in the methanogen community was a shift from *Methanomassiliicoccaceae* to *Methanosphera* in the wet season, which is likely linked to an increase in pectin from the diet.

The current findings and knowledge can help to implement new management tools such as the use of biomarkers (e.g., nitrogen fractionation or microbial signatures) and precision supplementation, thus alleviating the seasonal constraints on cattle grazing in dry tropical rangelands around the world.

## Figures and Tables

**Table 1:** Rumen fermentation parameters, body weight, ADWG, fecal N, BUN, and plasma nitrogen fractionation (δ ^15^N) in pregnant heifers grazing tropical forage at late-dry season, two months on supplementation or un-supplemented.

	Un-Supplemented	Supplemented	SEM	*p*-Value
Body weight (Kg)	379	437	8.17	0.002
N intake kg/day	0.002	0.023	0.001	0.001
Supplement intake kg/day	0.015	0.153	0.001	0.001
ADWG (Kg)	−0.374	0.674	0.09	0.001
δ ^15^N_plasma protein_	7.11	5.44	0.11	0.001
Fecal N %	1.10	1.12	0.03	0.72
Ammonia-N mg/L	14.3	23.9	3.17	0.15
BUN mg/100 mL	4.66	4.75	0.30	0.89
Total VFA mM	75.8	75.8	3.31	0.99
Fatty acid %				
Acetate	73.2	74.5	0.27	0.031
Propionate	18.2	14.0	0.38	0.005
Butyrate	7.70	8.65	0.16	0.007
iso-Butyrate	0.25	0.34	0.03	0.084
Valerate	0.37	0.41	0.01	0.167
iso-Valerate	0.24	0.32	0.03	0.161
Acetic:Propionic ratio	4.08	4.73	0.11	0.008

**Table 2:** Rumen fermentation parameters, body weight, fecal N, and BUN in heifers grazing at mid-dry and wet season (pregnant and lactating, respectively).

	Mid-Dry Season	Wet Season	SEM	*p*-Value
Body weight (Kg)	399	389	7.06	0.168
Fecal N %	0.97	2.12	0.03	0.001
Ammonia-N mg/L	11.9	70.2	2.34	0.001
BUN mg/100 mL	2.79	9.04	0.40	0.001
Total VFA mM	84.0	64.2	3.15	0.001
Fatty acid %				
Acetate	75.6	75.8	0.16	0.601
Propionate	14.1	12.0	0.13	0.001
Butyrate	8.99	9.37	0.07	0.085
iso-Butyrate	0.48	0.89	0.03	0.001
Valerate	0.34	0.64	0.01	0.001
iso-Valerate	0.44	1.09	0.03	0.001
Acetic:Propionic ratio	5.38	6.18	0.06	0.001

## Supplementary Materials

Supplementary materials can be found at https://www.mdpi.com/2076-2607/8/10/1550/s1. Table S1: Supplement nutrient composition, Table S2: Rumen fermentation parameters, body weight, ADWG, fecal N and BUN in pregnant heifers grazing tropical forage at late-dry season, prior supplementation, Table S3: Primers used for amplicon library preparation. Figure S1: Experiment timeline diagram, Figure S2: Alpha diversity measures for rumen bacteria (a), archaea (b), protozoa (c), and fungi (d) communities at un-supplemented and supplemented animals illustrating the Chao1 index (Chao1), Shannon diversity index (Shannon), and Simpson diversity index (Simpson), Figure S3: Supervised analysis with sPLS-DA on rumen bacteria (a), archaea (b), protozoa (c), and fungi (d) communities for un-supplemented and supplemented animals grazing in mid-dry, late-dry season, and wet season, Figure S4: Quantitative PCR analysis of rumen anaerobic fungi and protozoa population changes in response to supplementation in the late-dry season. t denotes a tendency (*p* < 0.1) of supplemented group compared to the un-supplemented group. The y-axis denotes fold change relative to un-supplemented animals’ fungi and protozoa populations respectively, Figure S5: Alpha diversity measures for rumen bacteria (a), archaea (b), protozoa (c), and fungi (d) communities from animals grazing in the mid-dry and wet seasons illustrating the Chao1 index (Chao1), Shannon diversity index (Shannon), and Simpson diversity index (Simpson), Figure S6: Quantitative PCR analysis of rumen anaerobic fungi and protozoa population changes in animals grazing at the mid-dry and wet season. *** Denote significant differences (*p* < 0.01) of wet season compared to the mid-dry season. The y-axis denotes fold change relative to the mid-dry season fungi and protozoa populations, respectively, Supplementary File S1: Bacterial ASVs significantly different (FDR < 0.05) between un-supplemented and supplemented animals in the late-dry season. Supplementary File S2: Archaeal ASVs significantly different (FDR < 0.05) between un-supplemented and supplemented animals in the late-dry season. Supplementary File S3: Bacterial ASVs significantly different (FDR < 0.05) between animals grazing in the mid-dry and wet seasons. Supplementary File S4: Archaeal ASVs significantly different (FDR < 0.05) between animals grazing in the mid-dry and wet seasons. Supplementary File S5: Protozoal ASVs significantly different (FDR < 0.05) between animals grazing in the mid-dry and wet seasons. Supplementary File S6: Fungal ASVs significantly different (FDR < 0.05) between animals grazing in the mid-dry and wet seasons.
