# Correction: Martinez-Fernandez et al. Seasonal and Nutrient Supplement Responses in Rumen Microbiota Structure and Metabolites of Tropical Rangeland Cattle. *Microorganisms* 2020, *8*, 1550

**DOI:** 10.3390/microorganisms9051053

**Published:** 2021-05-13

**Authors:** Gonzalo Martinez-Fernandez, Jinzhen Jiao, Jagadish Padmanabha, Stuart E. Denman, Christopher S. McSweeney

**Affiliations:** 1 Agriculture and Food, CSIRO, St Lucia, QLD 4067, Australia; gonzalo.martinezfernandez@csiro.au (G.M.-F.); Jagadish.Padmanabha@csiro.au (J.P.); stuart.denman@csiro.au (S.E.D.); 2 CAS Key Laboratory of Agro-Ecological Processes in Subtropical Region, Institute of Subtropical Agriculture, The Chinese Academy of Sciences, Changsha 410125, China; jjz@isa.ac.cn

We have found one inadvertent error in our paper published in *Microorganisms* [1]:

The authors wish to insert this additional sentence in the Materials and Methods Section 2.4: The sequences supporting the conclusions of this article are available in the European Nucleotide Archive repository under the accession numbers PRJEB39148 and PRJEB39175.

The authors wish to insert this additional sentence in the Acknowledgments section: We gratefully acknowledge Dr. G. Cantalapiedra-Hijar for his technical support, knowledge and assistance in analyzing and interpreting the natural abundance of nitrogen stable isotopes.

These changes have no material impact on the conclusions of our paper. We apologize to our readers.
