# Supplementary material for: Seasonal and Nutrient Supplement Responses in Rumen Microbiota Structure and Metabolites of Tropical Rangeland Cattle
Source: Microorganisms. 2020 Oct 8;8(10):1550. doi: 10.3390/microorganisms8101550 (PMC7600044; doi:10.3390/microorganisms8101550)
Supplement: Supplementary file 1 [file microorganisms-08-01550-s001.zip › Supplementary materials.docx]

**Table S1**. Supplement nutrient composition.

|  | Rumevite® |
| --- | --- |
| Total Crude Protein, % | 93 |
| Crude fat, % | 0.1 |
| Crude fibre, % | 10 |
| Urea, % | 30 |
| Phosphorous, % | 3.2 |
| Calcium, % | 7.0 |
| Fluorine, % | 0.1 |
| Sulphur, % | 2.2 |
| Copper, mg/kg | 300 |
| Cobalt, mg/kg | 30 |
| Iodine, mg/kg | 30 |
| Zinc, mg/kg | 500 |
| Selenium, mg/kg | 2.5 |
| Sodium, % | 12 |

**Table S2**. Rumen fermentation parameters, body weight, ADWG, faecal N and BUN in pregnant heifers grazing tropical forage at late-dry season, prior supplementation.

|  | Un-supplemented | Pre-supplemented | SEM | P-Value |
| --- | --- | --- | --- | --- |
| Body weight (Kg) | 401 | 398 | 6.47 | 0.813 |
| Faecal N (NIRS) % | 0.98 | 0.93 | 0.95 | 0.428 |
| Ammonia-N mg/L | 16.8 | 7.13 | 3.18 | 0.146 |
| BUN mg/100 mL | 2.81 | 2.77 | 0.29 | 0.907 |
| Total VFA mM | 82.14 | 85.9 | 3.46 | 0.592 |
| Fatty acid (mol/100 mol) |  |  |  |  |
| Acetate | 75.3 | 75.9 | 0.20 | 0.154 |
| Propionate | 14.2 | 14.0 | 0.19 | 0.573 |
| Butyrate | 9.18 | 8.82 | 0.09 | 0.069 |
| i-Butyrate | 0.25 | 0.34 | 0.02 | 0.330 |
| Valerate | 0.33 | 0.36 | 0.01 | 0.188 |
| i-Valerate | 0.45 | 0.43 | 0.03 | 0.763 |
| Acetic:Propionic | 5.30 | 5.44 | 0.08 | 0.412 |

**Table S3.** Primers used for amplicon library preparation.

| Targets | Forward primer |  | Reverse primer | | Product length (bp) | Literature |
| --- | --- | --- | --- | --- | --- | --- |
| Bacteria | 16Sf | GTGCCAGCMGCCGCGGTAA | 16Sr | GGACTACHVGGGTWTCTAAT | 250 | Kozich et al., 2013 |
| Archaea | Ar915aF | AGGAATTGGCGGGGGAGCAC | Ar1386R | GCGGTGTGTGCAAGGAGC | 492 | Watanabe et al., 2004; Skillman et al., 2004 |
| Fungi | MN100F | TCCTACCCTTTGTGAATTTG | MNGM2 | CTGCGTTCTTCATCGTTGCG | 250 | Tuckwell et al., 2005 |
| Protozoa | GIC1080F | GGGRAACTTACCAGGTCC | GIC1578R | GTGATRWGRTTTACTTRT | 498 | Ishaq et al., 2014 |
|  |  |  |  |  |  |  |

Ishaq SL, Wright AD. 2014. Design and validation of four new primers for next-generation sequencing to target the 18S rRNA genes of gastrointestinal ciliate protozoa. Appl Environ Microbiol 80:5515-21.

Kozich JJ, Westcott SL, Baxter NT, Highlander SK, Schloss PD. 2013. Development of a dual-index sequencing strategy and curation pipeline for analyzing amplicon sequence data on the MiSeq Illumina sequencing platform. Appl Environ Microbiol 79:5112-20.

Skillman LC, Evans PN, Naylor GE, Morvan B, Jarvis GN, et al. 2004. 16S ribosomal DNA-directed PCR primers for ruminal methanogens and identification of methanogens colonising young lambs. Anaerobe 10: 277–285.

Tuckwell DS, Nicholson MJ, McSweeney CS, Theodorou MK and Brookman JL. 2005. The rapid assignment of ruminal fungi to presumptive genera using ITS1 and ITS2 RNA secondary structures to produce group‐specific fingerprints. Microbiology 151:1557–1567.

Watanabe T, Asakawa S, Nakamura A, Nagaoka K, Kimura M. 2004. DGGE method for analyzing 16S rDNA of methanogenic archaeal community in paddy field soil. FEMS Microbiology Letters 232: 153–163.

**
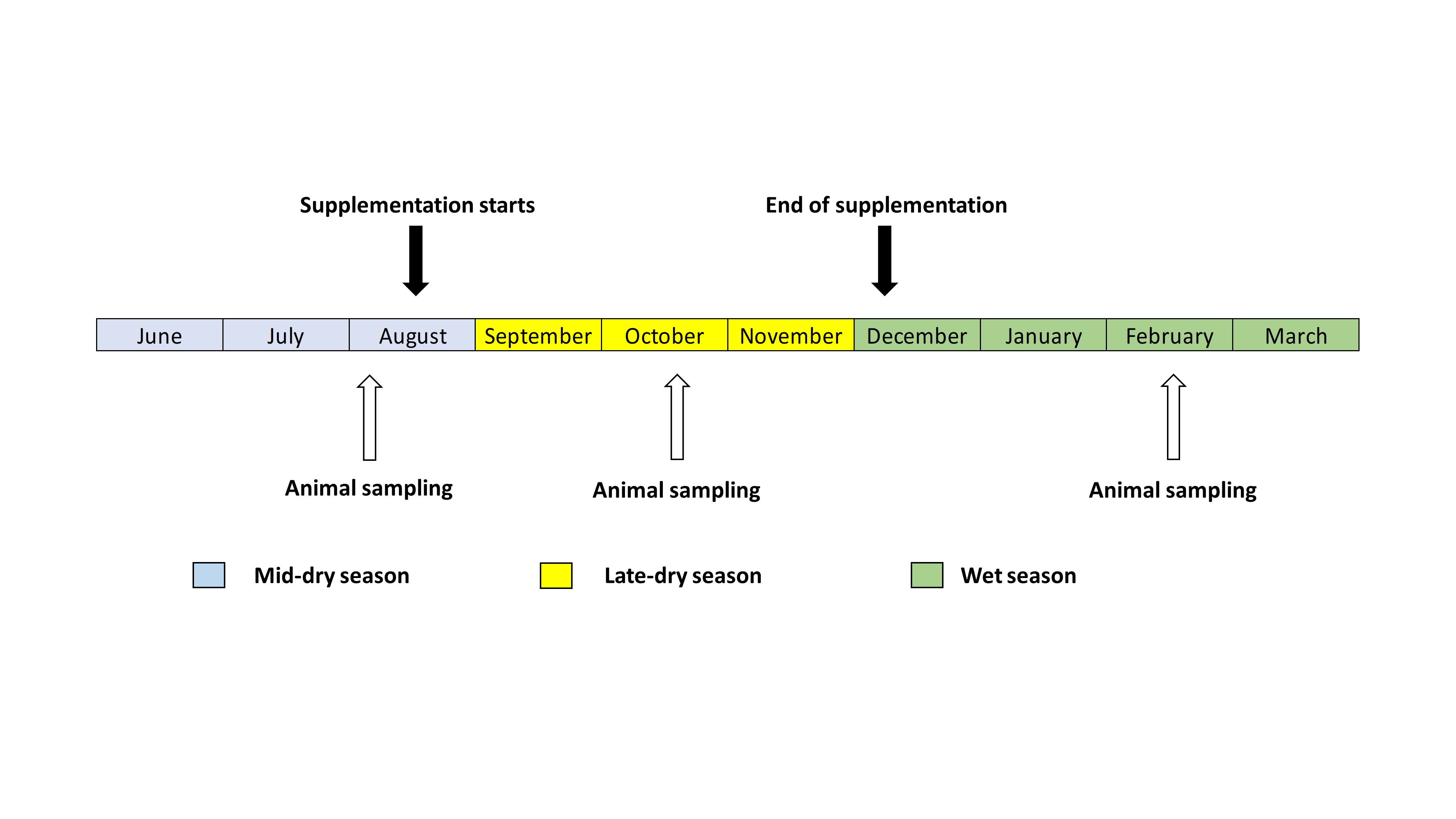
**

**Figure S1.** Experiment timeline diagram.


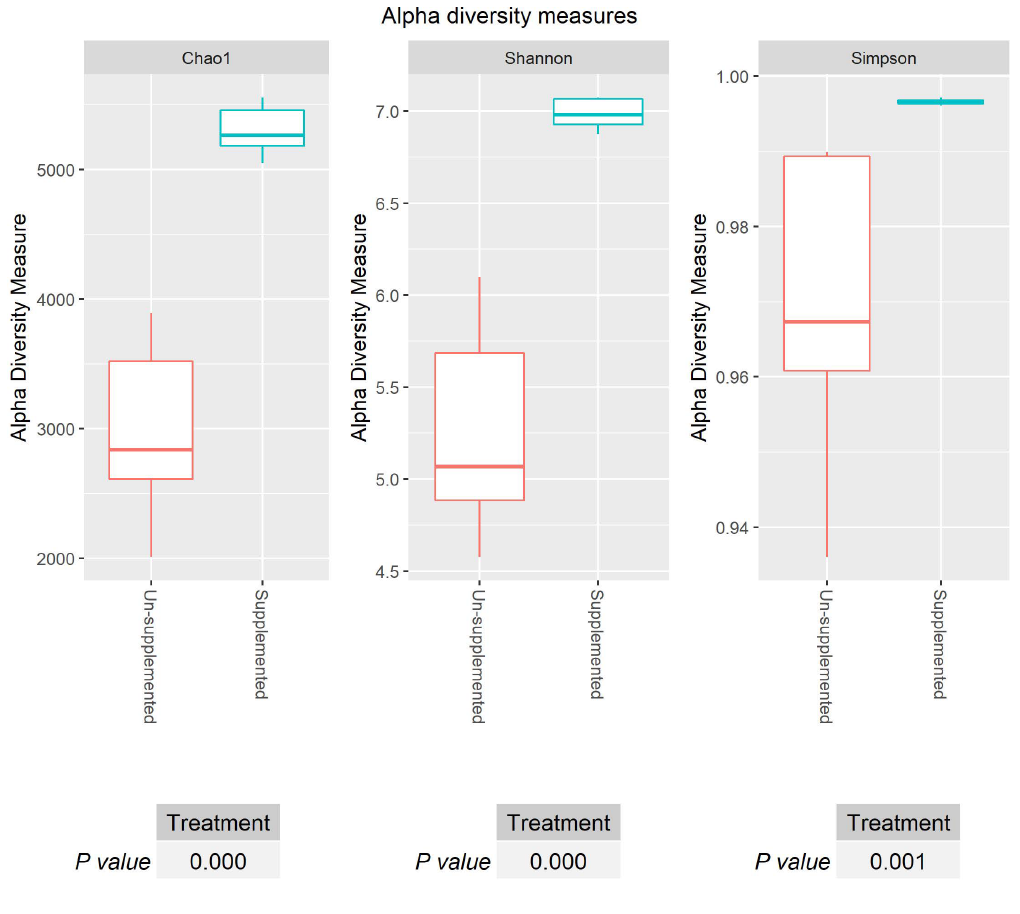

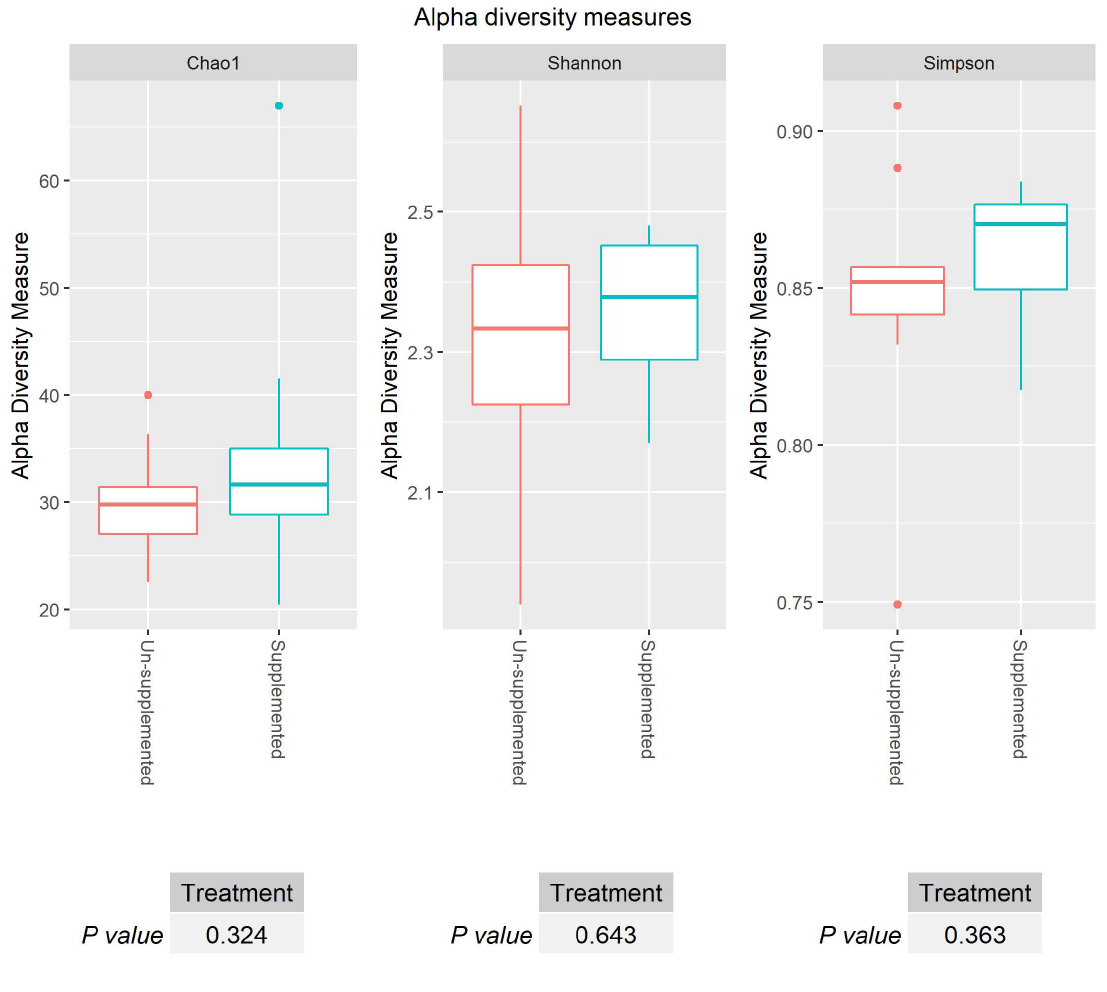


(b)

(a)


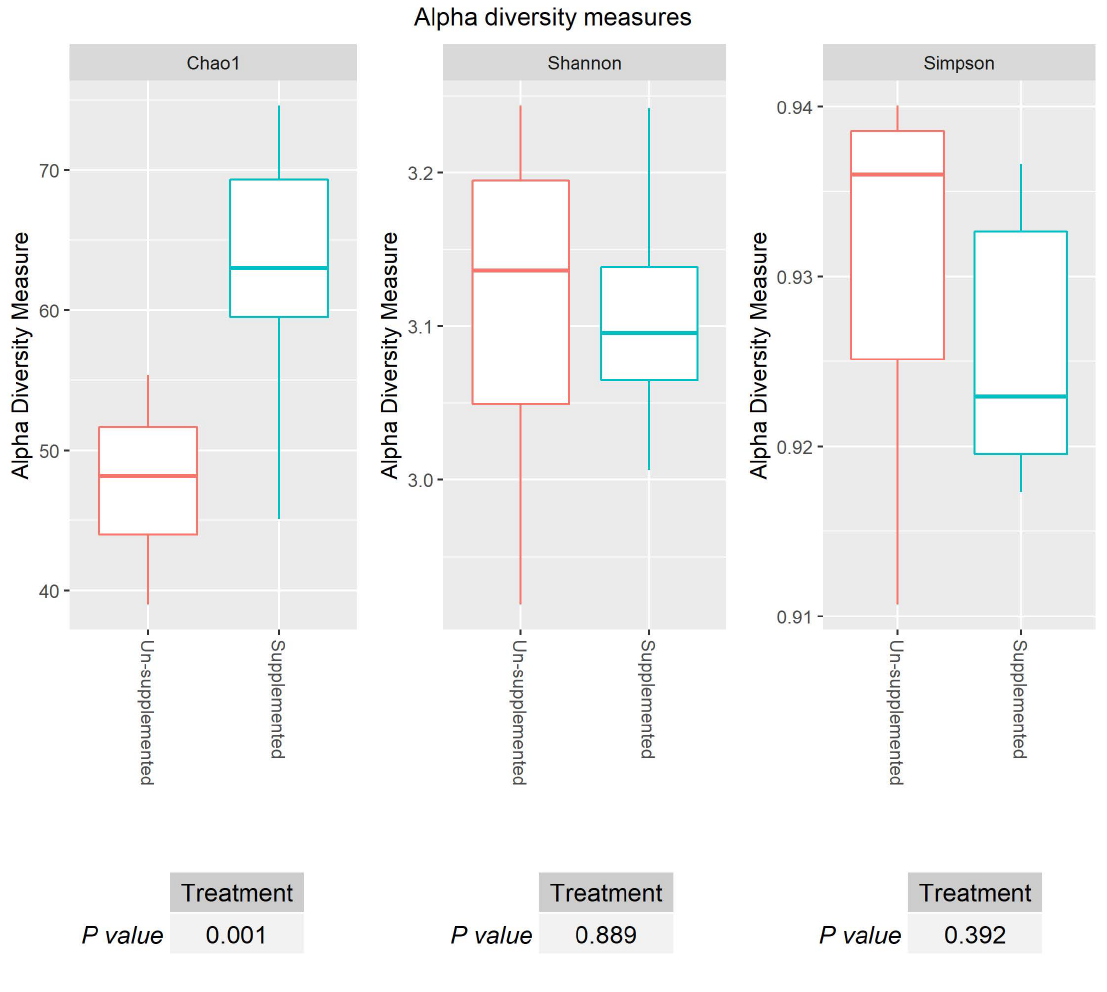

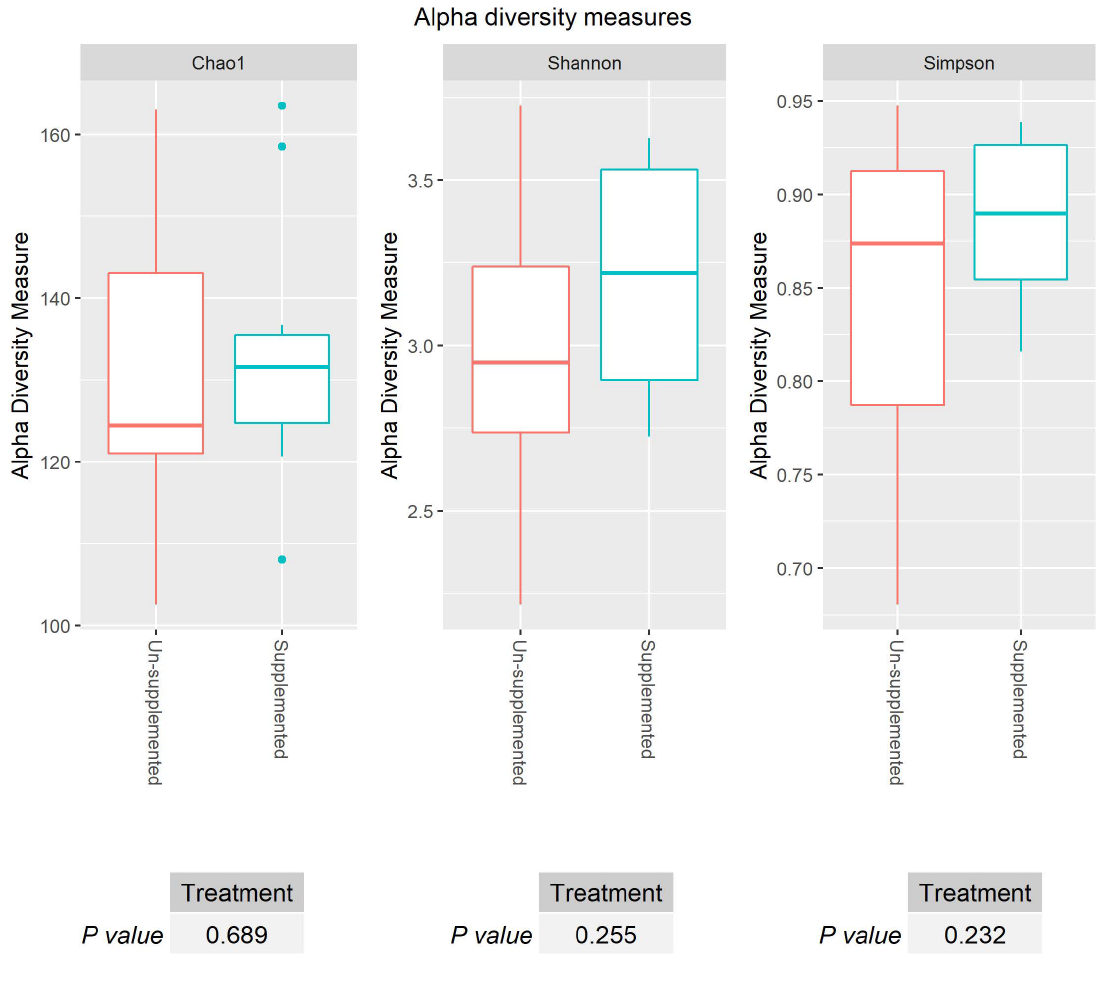


(d)

(c)

**Figure S2.** Alpha diversity measures for rumen bacteria (a), archaea (b), protozoa (c) and fungi (d) communities at un-supplemented and supplemented animals illustrating the Chao1 index (Chao1), Shannon diversity index (Shannon) and Simpson diversity index (Simpson). Boxplots indicate variance within the sampled animals with the box boundaries showing the first and third quartiles, the median value indicated as a horizontal line and the whiskers extend to 1.5 times the interquartile range.


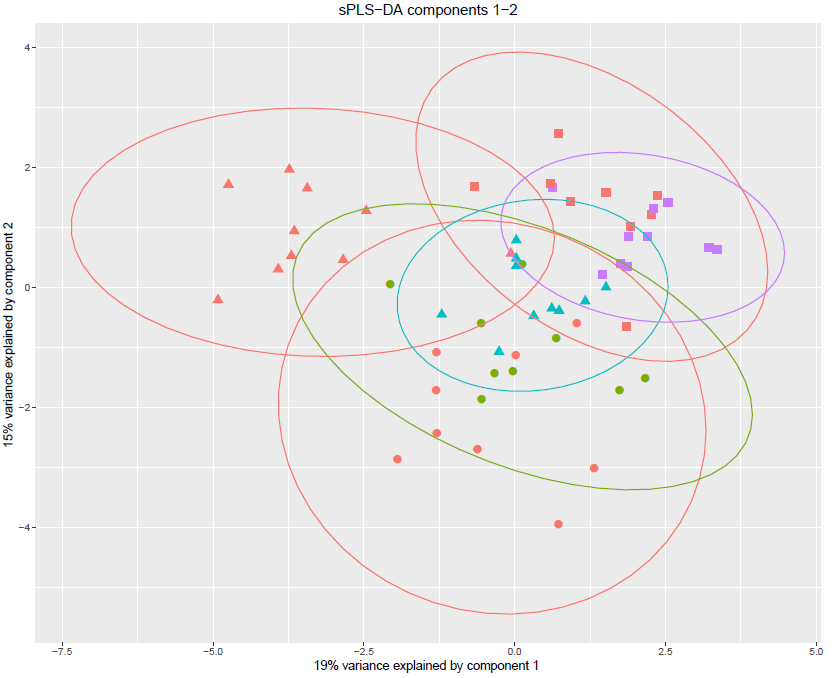

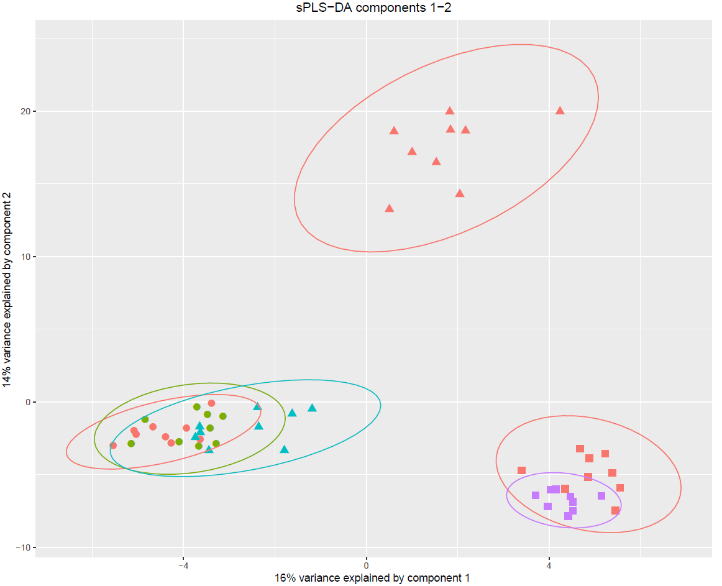


(b)

(a)


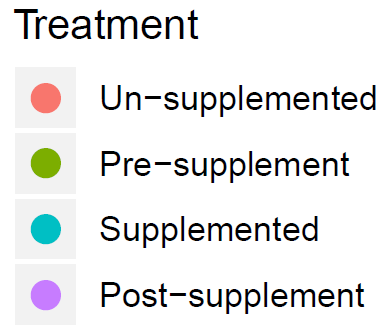


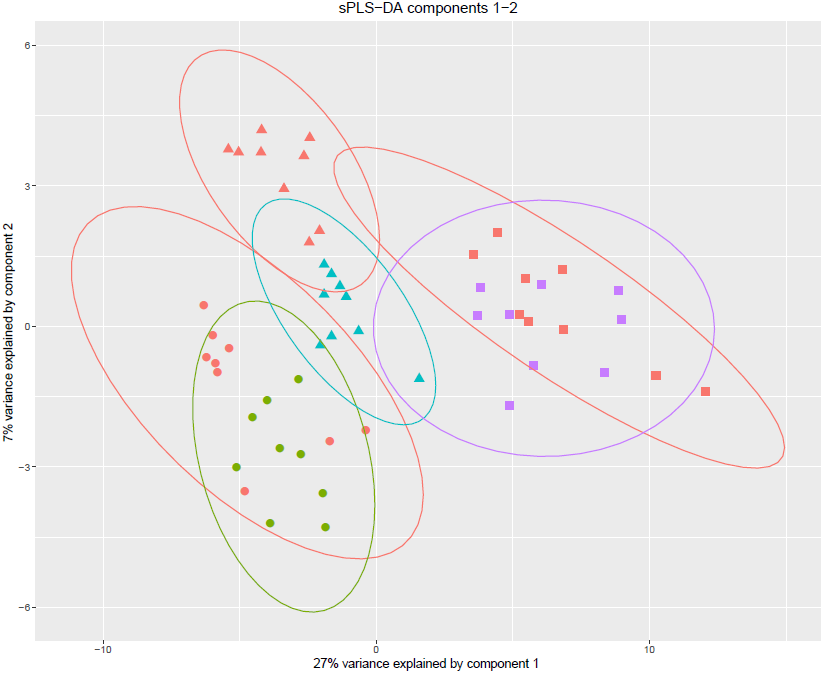

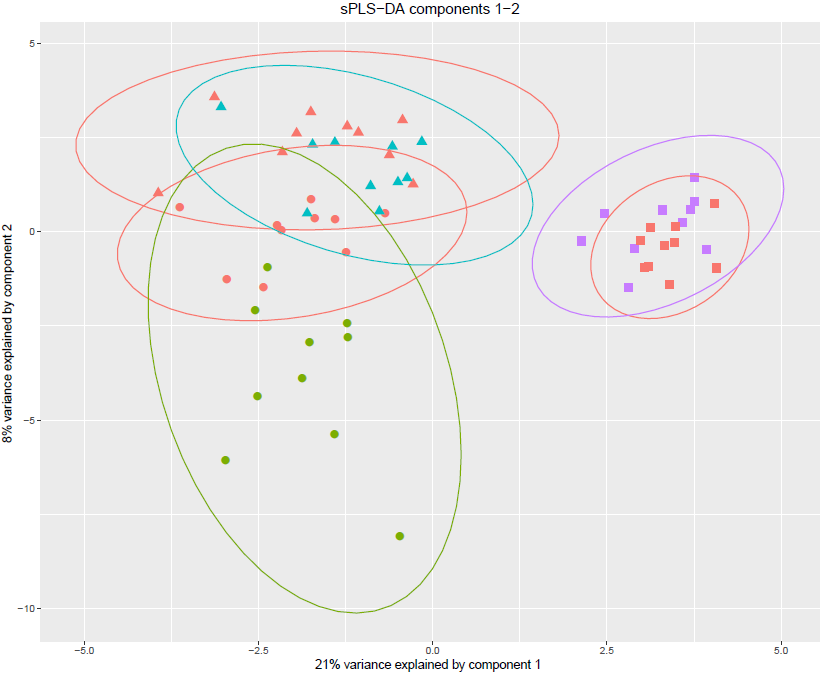

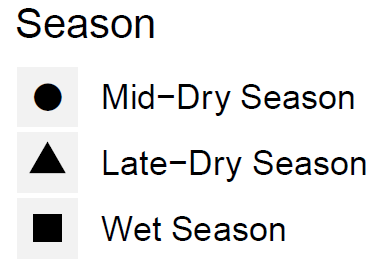


(d)

(c)

**Figure S3.** Supervised analysis with sPLS-DA on rumen bacteria (a), archaea (b), protozoa (c) and fungi (d) communities for un-supplemented and supplemented animals grazing in mid-dry, late-dry season and wet season

**Figure S4.** Quantitative PCR analysis of rumen anaerobic fungi and protozoa population changes in response to supplementation in the late-dry season. ***^t^*** denotes a tendency (P < 0.1) of supplemented group compared to the un-supplemented group. The y-axis denotes fold change relative to un-supplemented animals’ fungi and protozoa populations respectively.


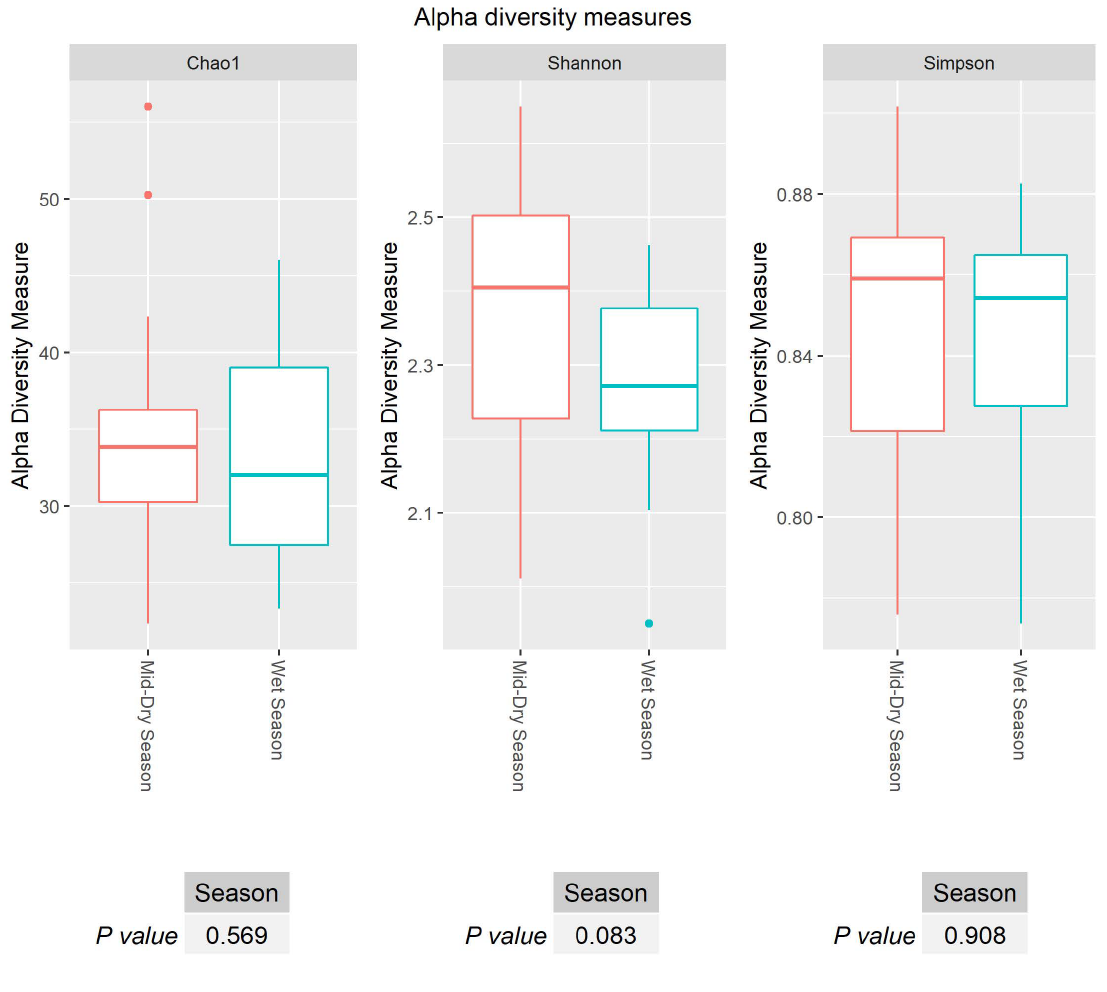

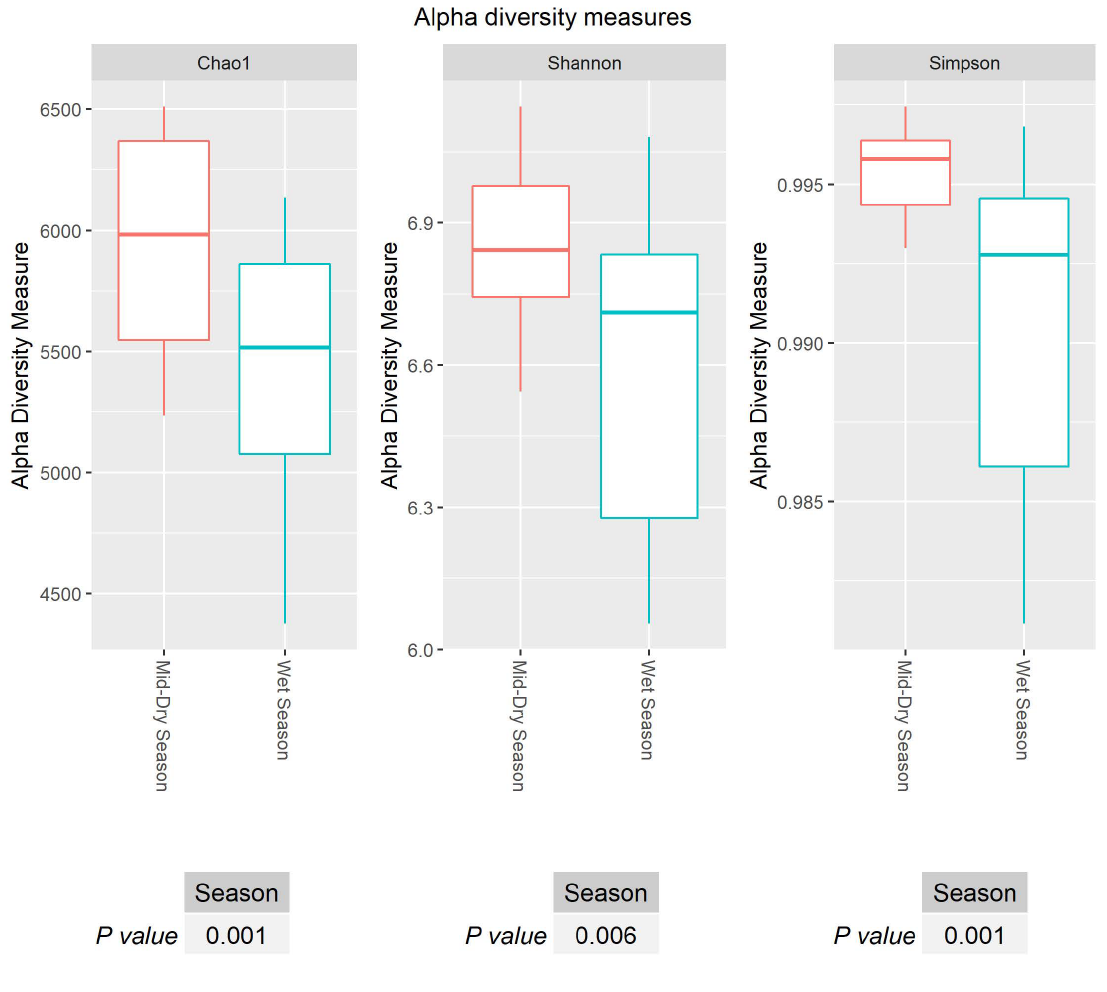

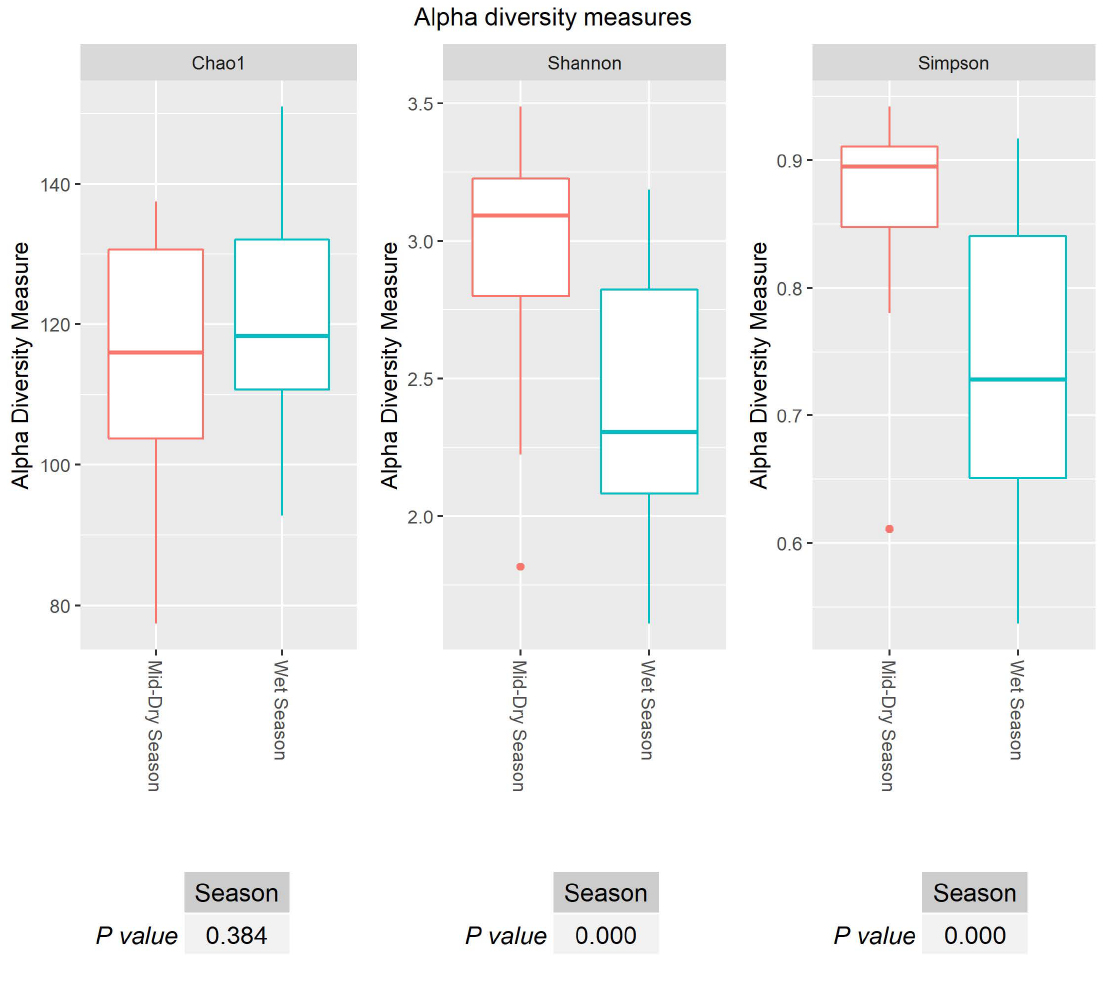

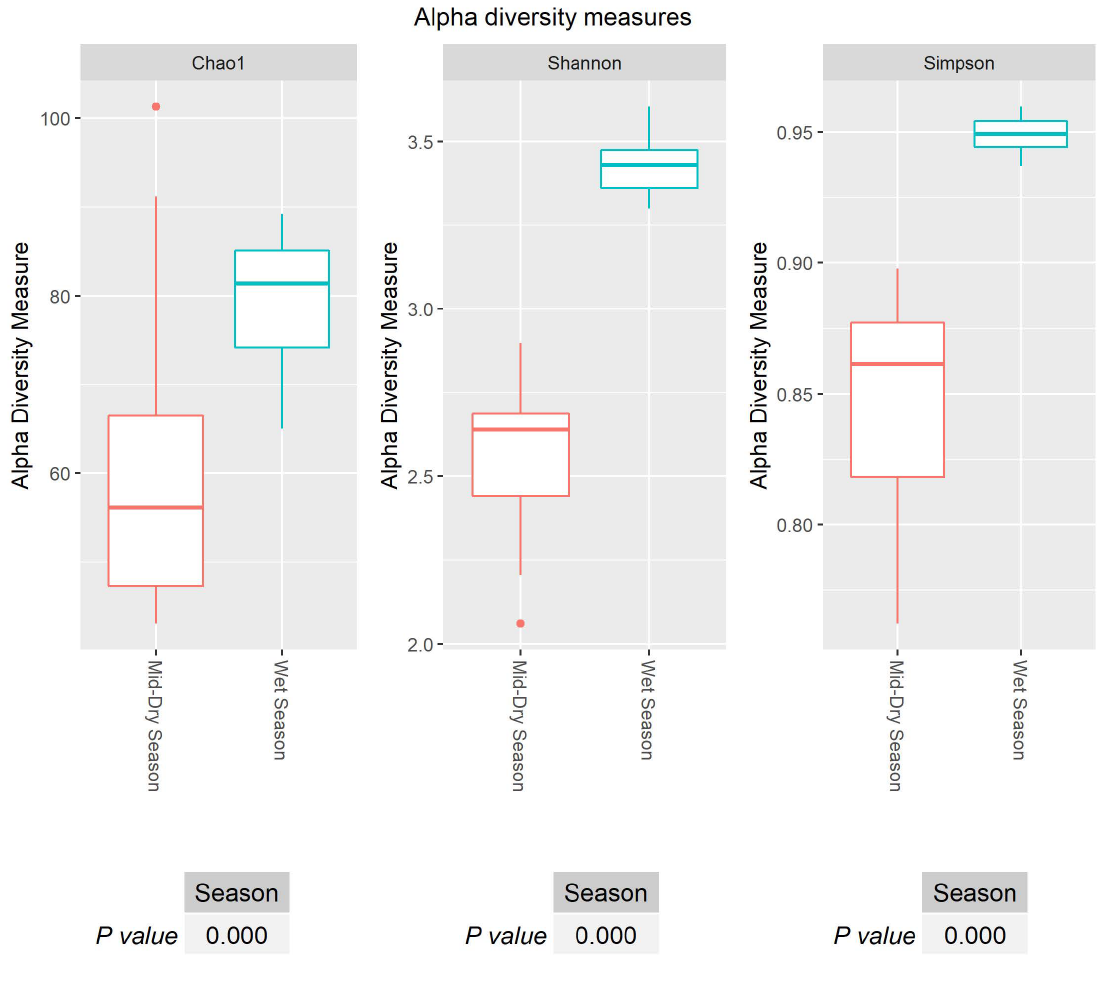


(d)

(c)

(b)

(a)

**Figure S5.** Alpha diversity measures for rumen bacteria (a), archaea (b), protozoa (c) and fungi (d) communities from animals grazing in the mid-dry and wet seasons illustrating the Chao1 index (Chao1), Shannon diversity index (Shannon) and Simpson diversity index (Simpson). Boxplots indicate variance within the sampled animals with the box boundaries showing the first and third quartiles, the median value indicated as a horizontal line and the whiskers extend to 1.5 times the interquartile range.

**Figure S6.** Quantitative PCR analysis of rumen anaerobic fungi and protozoa population changes in animals grazing at the Mid-Dry and wet season. *** denote significant differences (P < 0.01) of wet season compared to the mid-dry season. The y-axis denotes fold change relative to the mid-dry season fungi and protozoa populations respectively.
